# Radiomics workflow definition & challenges - German priority program 2177 consensus statement on clinically applied radiomics

**DOI:** 10.1186/s13244-024-01704-w

**Published:** 2024-06-03

**Authors:** Ralf Floca, Jonas Bohn, Christian Haux, Benedikt Wiestler, Frank G. Zöllner, Annika Reinke, Jakob Weiß, Marco Nolden, Steffen Albert, Thorsten Persigehl, Tobias Norajitra, Bettina Baeßler, Marc Dewey, Rickmer Braren, Martin Büchert, Eva Maria Fallenberg, Norbert Galldiks, Annika Gerken, Michael Götz, Horst K. Hahn, Johannes Haubold, Tobias Haueise, Nils Große Hokamp, Michael Ingrisch, Andra-Iza Iuga, Marco Janoschke, Matthias Jung, Lena Sophie Kiefer, Philipp Lohmann, Jürgen Machann, Jan Hendrik Moltz, Johanna Nattenmüller, Tobias Nonnenmacher, Benedict Oerther, Ahmed E. Othman, Felix Peisen, Fritz Schick, Lale Umutlu, Barbara D. Wichtmann, Wenzhao Zhao, Svenja Caspers, Heinz-Peter Schlemmer, Christopher L. Schlett, Klaus Maier-Hein, Fabian Bamberg

**Affiliations:** 1 German Cancer Research Center (DKFZ) Heidelberg, Division of Medical Image Computing, Heidelberg, Germany; 2 Pattern Analysis and Learning Group, Department of Radiation Oncology, Heidelberg University Hospital, Heidelberg, Germany; 3 National Center for Radiation Research in Oncology NCRO, Heidelberg Institute for Radiation Oncology HIRO, Heidelberg, Germany; 4 Faculty of Bioscience, University of Heidelberg, Heidelberg, Germany; 5 National Center for Tumor Diseases (NCT), NCT Heidelberg, a partnership between DKFZ and University Medical Center Heidelberg, Heidelberg, Germany; 6 Translational Lung Research Center (TLRC), German Center for Lung Research (DZL), Heidelberg, Germany; 7 Department of Radiation Oncology, Heidelberg University Hospital, Heidelberg, Germany; 8 Department of Neuroradiology, TU Munich University Hospital, Munich, Germany; 9 TranslaTUM - Central Institute for Translational Cancer Research, TU Munich, Munich, Germany; 10 Computer Assisted Clinical Medicine, Medical Faculty Mannheim, Heidelberg University, Heidelberg, Germany; 11 Mannheim Institute for Intelligent Systems in Medicine, Medical Faculty Mannheim, Heidelberg University, Heidelberg, Germany; 12 Intelligent Medical Systems, German Cancer Research Center (DKFZ), Heidelberg, Germany; 13 Helmholtz Imaging, German Cancer Research Center (DKFZ), Heidelberg, Germany; 14 Department of Diagnostic and Interventional Radiology, Medical Center, Faculty of Medicine Freiburg, University of Freiburg, Freiburg, Germany; 15 Institute for Diagnostic and Interventional Radiology, Faculty of Medicine and University Hospital Cologne, University Cologne, Cologne, Germany; 16 Department of Diagnostic and Interventional Radiology, University Hospital Würzburg, Würzburg, Germany; 17 Charité — Universitätsmedizin Berlin, corporate member of Freie Universität Berlin and Humboldt-Universität zu Berlin, Department of Radiology, Berlin Institute of Health, DZHK (German Centre for Cardiovascular Research), and DKTK (German Cancer Consortium), both partner sites Berlin, Berlin, Germany; 18 Institute of Diagnostic and Interventional Radiology, Technical University of Munich, School of Medicine & Health, Ismaninger Str. 22, 81675 München, Germany; 19 Artificial Intelligence in Healthcare and Medicine, School of Computation, Information and Technology, Technical University of Munich, Munich, Germany; 20 German Cancer Consortium (DKTK), Munich partner site, Heidelberg, Germany; 21 Department of Neurology, Faculty of Medicine and University Hospital Cologne, Cologne, Germany; 22 Institute of Neuroscience and Medicine (INM-3), Research Center Juelich (FZJ), Juelich, Germany; 23 Center of Integrated Oncology Aachen Bonn Cologne Duesseldorf (CIO ABCD), Aachen, Bonn, Cologne & Duesseldorf, Germany; 24 Fraunhofer Institute for Digital Medicine MEVIS, Bremen, Germany; 25 Division of Experimental Radiology, Department for Diagnostic and Interventional Radiology, University Hospital Ulm, Ulm, Germany; 26 Faculty 3, Mathematics and Computer Science, University of Bremen, Bremen, Germany; 27 Department of Diagnostic and Interventional Radiology and Neuroradiology, University Hospital Essen, Essen, Germany; 28 Section on Experimental Radiology, Department of Diagnostic and Interventional Radiology, University Hospital Tübingen, Tübingen, Germany; 29 Institute for Diabetes Research and Metabolic Diseases (IDM) of the Helmholtz Center Munich at the University of Tübingen, Tübingen, Germany; 30 German Center for Diabetes Research (DZD), Tübingen, Germany; 31 Department of Radiology, University Hospital, LMU Munich, Munich, Germany; 32 Department of Diagnostic and Interventional Radiology, University Hospital Tübingen, Tübingen, Germany; 33 Department of Nuclear Medicine and Clinical Molecular Imaging, University Hospital Tübingen, Tübingen, Germany; 34 Institute of Neuroscience and Medicine (INM-4), Research Center Juelich (FZJ), Juelich, Germany; 35 Department of Nuclear Medicine, University Hospital RWTH Aachen, Aachen, Germany; 36 Department of Diagnostic and Interventional Radiology, University Hospital Heidelberg, Heidelberg, Germany; 37 Department of Neuroradiology, University Medical Center, Johannes Gutenberg University Mainz, Mainz, Germany; 38 Department of Diagnostic and Interventional Radiology, University Hospital Bonn, Bonn, Germany; 39 Institute of Neuroscience and Medicine (INM-1), Research Centre Jülich, Jülich, Germany; 40 Institute for Anatomy I, Medical Faculty, Heinrich Heine University Düsseldorf, Düsseldorf, Germany; 41 German Cancer Research Center (DKFZ) Heidelberg, Division of Radiology, Heidelberg, Germany

**Keywords:** Image processing, Computer-assisted, Workflow, Terminology, Consensus development conference

## Abstract

**Objectives:**

Achieving a consensus on a definition for different aspects of radiomics workflows to support their translation into clinical usage. Furthermore, to assess the perspective of experts on important challenges for a successful clinical workflow implementation.

**Materials and methods:**

The consensus was achieved by a multi-stage process. Stage 1 comprised a definition screening, a retrospective analysis with semantic mapping of terms found in 22 workflow definitions, and the compilation of an initial baseline definition. Stages 2 and 3 consisted of a Delphi process with over 45 experts hailing from sites participating in the German Research Foundation (DFG) Priority Program 2177. Stage 2 aimed to achieve a broad consensus for a definition proposal, while stage 3 identified the importance of translational challenges.

**Results:**

Workflow definitions from 22 publications (published 2012–2020) were analyzed. Sixty-nine definition terms were extracted, mapped, and semantic ambiguities (e.g., homonymous and synonymous terms) were identified and resolved. The consensus definition was developed via a Delphi process. The final definition comprising seven phases and 37 aspects reached a high overall consensus (> 89% of experts “agree” or “strongly agree”). Two aspects reached no strong consensus. In addition, the Delphi process identified and characterized from the participating experts’ perspective the ten most important challenges in radiomics workflows.

**Conclusion:**

To overcome semantic inconsistencies between existing definitions and offer a well-defined, broad, referenceable terminology, a consensus workflow definition for radiomics-based setups and a terms mapping to existing literature was compiled. Moreover, the most relevant challenges towards clinical application were characterized.

**Critical relevance statement:**

Lack of standardization represents one major obstacle to successful clinical translation of radiomics. Here, we report a consensus workflow definition on different aspects of radiomics studies and highlight important challenges to advance the clinical adoption of radiomics.

**Key Points:**

- Published radiomics workflow terminologies are inconsistent, hindering standardization and translation.
- A consensus radiomics workflow definition proposal with high agreement was developed.
- Publicly available result resources for further exploitation by the scientific community.

**Graphical Abstract:**

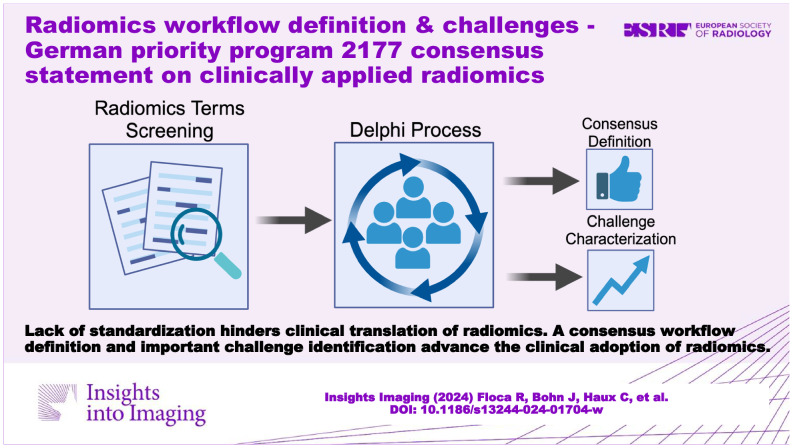

## Introduction

Substantial biomedical and technological progress during the past decades in capturing health-related characteristics such as molecular, genetic, metabolic, or morphological traits has facilitated increasingly personalized approaches towards disease management [1]. A key to personalized medicine is the detection of discriminating trait constellations, which may for instance be provided by imaging modalities such as computed tomography, magnetic resonance imaging, or positron emission tomography [2–5]. By exploiting software-based image analysis, multiple pattern extraction, and large-scale bioinformatics correlation analyses, advanced image post-processing and interpretation approaches (often, including this publication, also subsumed under the term ‘radiomics’) may allow for a more comprehensive image analysis [6] and trait detection.

However, while radiomics-derived imaging biomarkers may provide new insights, their traditional clinical role is merely limited to providing crude information such as the size, shape, or density of apparent disease processes. Thus, despite significant recent research efforts and accumulating evidence of their value for diagnostic, therapeutic, prognostic, and preventive schemes, these approaches have not been widely implemented into clinical workflows and radiological services yet [7].

The lack of translation of radiomics research into practical clinical applications can be attributed to various factors and still exists, despite existing initiatives such as the image biomarker standardization initiative (IBSI) [8], Radiomics Quality Score (RQS) [7], Quantitative Imaging Biomarker Alliance (QIBA; https://www.rsna.org/research/quantitative-imaging-biomarkers-alliance), CheckList for Evaluation of Radiomics Research (CLEAR) [9], Assessment of Radiomics research (ARISE) [10], and guideline framing. Some studies, like CLEAR and ARISE, provide essential checklists aimed at ensuring thorough and reproducible reporting in radiomics research. One reason for this still existing lack is the absence of a unified set of common definitions of workflow terms to ensure comparability and correct classification of workflows. It is hindering, i.a., correct application of guidelines (like RQS) or comparison/reproducibility of experimental setups and therefore ultimately successful clinical translation. Therefore, the need for collaborative efforts within the scientific community to establish such a consensus terminology becomes apparent in addressing these challenges.

To address this within the framework of the German Research Foundation (DFG, Deutsche Forschungsgemeinschaft) Priority Program “Radiomics”, we analyzed existing workflow definitions and conducted a Delphi process [11] to achieve the following: (i) a semantic analysis and mapping of existing definitions, (ii) a proposal for a workflow definition with high consensus to improve comparability and explainability of workflows, and (iii) an identification of the most important challenges that currently hinder the translation of such workflows into clinical routine.

## Methods

### Study design

**Fig. 1:**
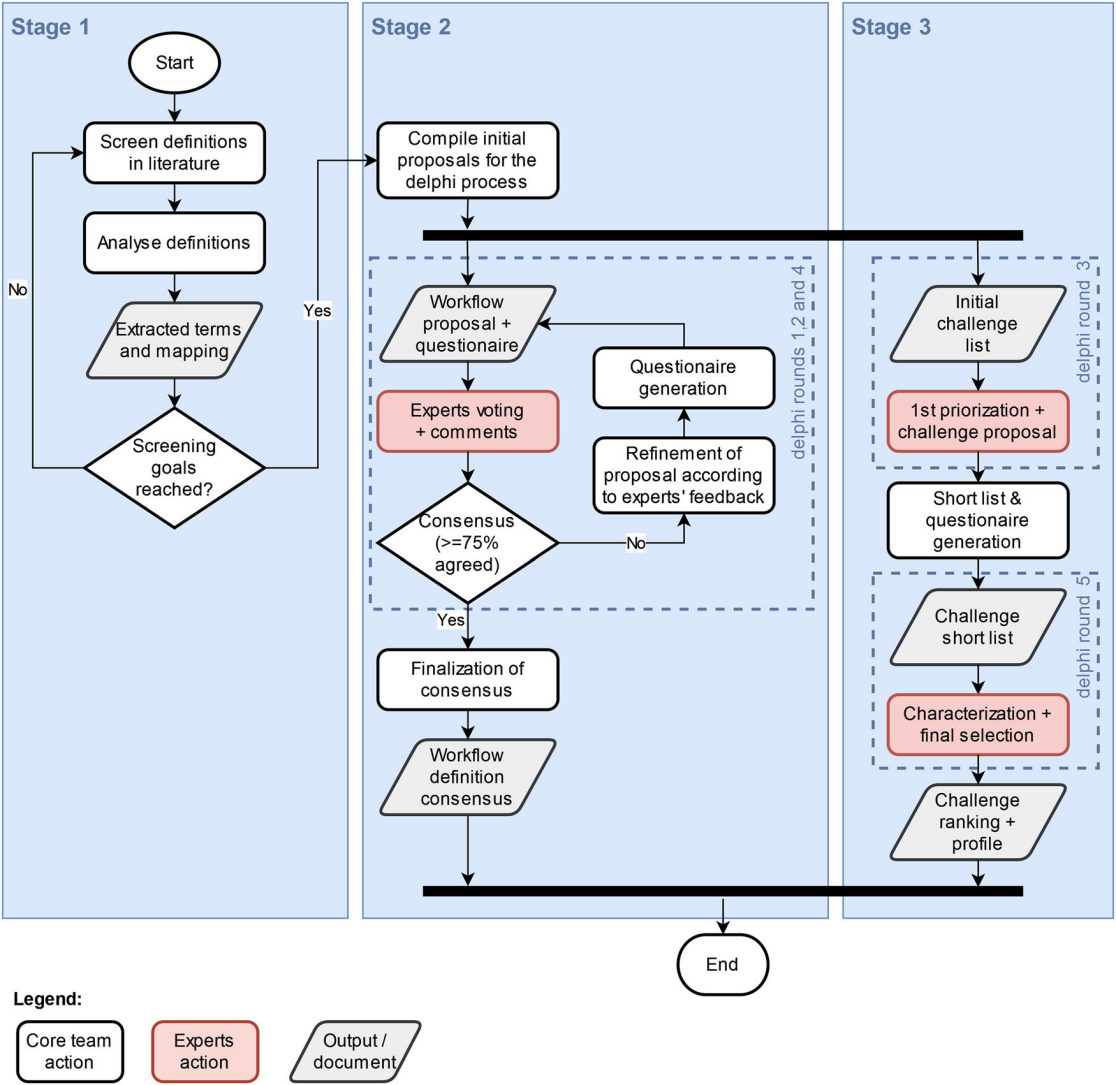
Flowchart depicting all steps of the study from preparation (stage 1) to the Delphi process (stage 2). The different rounds of the Delphi process are also indicated (blue dashed line boxes). The actions taken by the experts panel in the Delphi process are marked by red boxes

This study was divided into three stages (see Fig. 1). In a retrospective definition screening (stage 1) we collected workflow items and terminologies used in published radiomics studies as well as reported translational challenges to establish a starting point for the consensus-building Delphi process (stages 2 & 3, prospective). In this Delphi process, domain experts (for details see “DFG Priority Program 2177 Radiomics” below) rated the workflow items and refined the terminology towards a consensus (stage 2). In a third (prospective) study stage the challenges were characterized and ranked by our experts using the same Delphi process as in stage 2. The details of each stage are given in the following.

### Definition screening and analysis (stage 1)

A definition screening was conducted with two screening goals: (i) determine the existence of controversial/ambiguous definitions and (ii) provide input for the baselines of the Delphi process. Its search strategy was as follows. A PubMed search was conducted using the search string *“radiomic”[All Fields] OR “radiomics”[All Fields]* and Best-Match sorting. Furthermore, two queries were made to the Google search engine using (i) the term “radiomics”, and (ii) the terms “radiomics” and “FAIR” to see if radiomics standardization approaches exist in the context of FAIR principles (www.go-fair.org). In addition, reference sections in the retrieved publications as well as similar publications suggested by PubMed were reviewed. The searches were conducted on February 15, 2021 with no filters to narrow the search. Publications were included if they provided relevant content (workflow definitions or challenges). The inclusion was stopped by the core team when enough content was extracted to find evidence for controversial/ambiguous definitions and provide input for a baseline definition.

The included publications were examined for text passages that mentioned steps of a radiomics workflow. A coding system was created inductively from the text passages, by conducting the text research and building a terminology using the software MAXQDA 2020 [12], a widespread tool for qualitative data analysis. A new category was created for each newly named workflow step in the initial version of the coding system. Subsequently, an initial draft for a radiomics workflow was created based on the extracted steps (see supplement 1). In this process, all steps were mapped into a semantic hierarchy (including synonymous and homonymous steps).

### Workflow definition consensus process (stage 2)

A consensus decision was derived utilizing a structured Delphi process [11], aiming to achieve an agreement for a specific topic among a panel of experts. A Delphi process comprises several rounds in which a core team presents several hypotheses or assumptions in the form of questionnaires to the expert panel which are then voted upon. The core team members are exempted from the votes. The feedback from the panel is incorporated by the core team and made transparent to the experts in the following rounds, in which the process is repeated. The assumptions are thus incrementally refined based on the expert agreement (measured on a 5-level Likert scale) until a consensus is reached. In this study, consensus was reached if at least 75% of the experts agreed.

The Delphi process in this study was composed of five rounds of questionnaires. Three rounds (rounds 1, 2 and 4) focused on resolving terminology conflicts and achieving a consensus definition for different aspects of a radiomics workflow. The process began with an initial definition proposal derived from the definition screening and analysis results.

### Challenge characterization process (stage 3)

The aforementioned Delphi process was also used to identify the most important current roadblocks to the clinical translation of radiomics workflows.

Two rounds (rounds 3 and 5) of this process focused on achieving consensus about the importance of different challenges and on establishing a first characterization. The priority was deduced by (i) allowing each expert to make a priority selection and (ii) evaluating the frequency with which each challenge was picked. The baseline was a list of 32 challenges mentioned in the screened literature. Round 3 involved selection from the literature-based challenges or a proposal of additional challenges (up to seven prioritized challenges in total). Round 5 involved the (i) selection of up to three challenges from a shortlist (top ten literature-based and four expert-proposed challenges) and (ii) the characterization of the shortlisted challenges.

### DFG priority program 2177 radiomics

The Priority Program (SPP) 2177 includes 16 different projects with more than 45 experts from the interdisciplinary field of radiomics and is funded by the DFG to advance the diagnostic and prognostic value of medical imaging by implementing radiomics (including advanced image interpretation approaches such as deep learning algorithms) in different clinical scenarios (https://gepris.dfg.de/gepris/projekt/402688427?language=en). The program provides national, coordinated, competitive funding for and rigorous selection of independent research projects within its scientific objective coming from 19 research institutes of 15 locations in Germany. It creates added value by fostering collaboration among different disciplines and locations. As such, it provides a unique formation of national experts in the field of radiomics and is used in this study for fostering standardizations and problem statements to support clinical translation.

The experts for the Delphi process were recruited from the projects participating in SPP 2177. There were no other selection criteria for experts than their affiliation with a SPP 2177 radiomics project. For each round, invitations for participation were sent out to all project teams. The participation was voluntary and it was possible to participate anonymously.

### Availability of data and materials

The survey data conducted during the current study are available in the RadiomicsOntologySPP repository, https://github.com/MIC-DKFZ/radiomics-workflow-definition.

## Results

### Participating experts

**Fig. 2:**
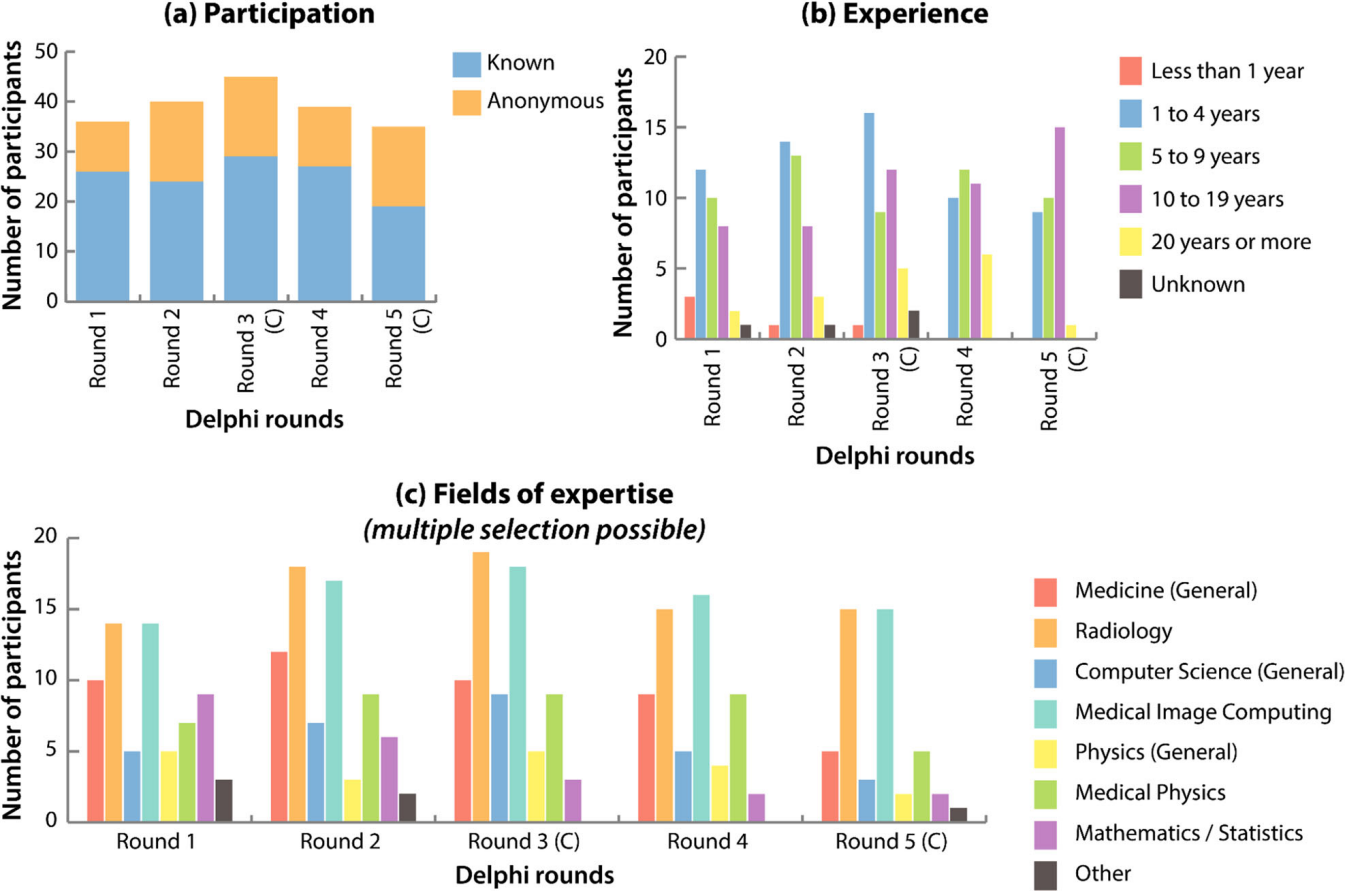
**a** Numbers of participants (separated in known participants and anonymous participants) over the course of the Delphi process (Delphi rounds). **b** Experience (in years) of participants over the course of the Delphi rounds. **c** Representation of fields of expertise over the course of the Delphi rounds. Multiple selections of fields of expertise per participant were possible. Rounds marked with a “(C)” (rounds 3 and 5) were rounds that focused on the challenge prioritization and characterization

Over the course of the Delphi process, on average 39 experts (standard deviation +/− 3.5) participated per round and 45 named experts participated at least in one round. As anonymous participation was possible, the total number of participating individual experts cannot be determined. Figure 1 depicts the flow chart of the study including the Delphi process and Fig. 2 shows the overall participation trend throughout the process. The topic-related working experience of all participating experts ranges from less than one year to up to 20 or more years. For the Delphi process, the percentage of senior experts (5 years and more of experience) ranged from 56% to 74% (see Fig. 2).

The field of expertise of the participants can be grouped into medicine (including radiology), computer science (including medical image computing), physics (including medical physics) and mathematics/statistics. The two most represented fields were medicine (ranging from 36% to 42%) followed by computer science (ranging from 28% to 38%); for further details and trends see Fig. 2.

### Definition screening and analysis (stage 1)

A total of 51 publications were screened for relevant content: 30 publications from PubMed; 5 publications from Google search and 16 publications from searching in references, supplemental material, and similar studies. Radiomics workflow definitions were found in 22 publications [7, 8, 13–32], and 95 workflow step terms were extracted (for details see supplement 1 - List of extracted step terms and mapping**)**. The most frequently mentioned terms are listed in Table 1.

**Table 1:** Most frequent workflow step terms found in the Radiomics workflow definition screening (see supplement 1-List of extracted step terms and mapping)

#	Term	Frequency
1	Feature extraction	12
2	Image acquisition^a^	12
3	Segmentation	9
4	Feature selection	9
5	Validation	6
6	Image segmentation	4
7	Analysis	4
8	Reconstruction	3
9	Modeling	3

Column “Frequency” displays the number of publications that used the given term. In total, we analyzed 22 publications that define Radiomics workflow terminology

^a^The term “Image acquisition” was used with two different semantics. Eight times it was used for data acquisition in general; encompassing not just the images but all data needed. Four times the term was used just for the acquisition of images

Forty-five conflicts concerning synonyms, homonyms, hierarchy, and semantic ambiguity were detected during validation (see supplement 1). Synonyms occurred when different terms were used for the same step (e.g., “feature calculation” or “quantification” for “feature extraction”). Homonyms were found when identically named steps were defined differently (e.g., “ROI extraction” in Murray et al [25] corresponds to “segmentation”, whereas in Zwanenburg et al [32] it corresponds to a substep of “feature extraction”). Hierarchy conflicts occurred when a step was mentioned as a main step in one publication, while it was a substep in another publication (e.g., “model building” in Avanzo et al [13] was identical to the main step “modeling”, whereas “model building” in Ibrahim et al [19], Lee et al [21], and Yang et al [31] was identified to be a substep of “modeling”). Semantic ambiguities occurred where definitions could not be clearly assigned to a step (e.g., “choice of imaging protocol” is described in Lambin et al [7] as possibly being a substep of both “data selection” and “data acquisition”). After creating a hierarchy and addressing the conflicts, a baseline for the consensus was modeled. This generic radiomics workflow consists of eight main steps and 28 substeps, called phases and aspects throughout the Delphi process and the results (see supplement 1).

### Workflow definition consensus (stage 2)

The consensus version of the workflow definition presented here was structured as follows: the top-level consists of up to seven phases (study design; data acquisition; data management; image processing and segmentation; feature extraction; modeling; reporting). Phases represent different fundamental workflow steps and can therefore, to a certain extent, be found in every radiomics workflow. Between most phases, there is a logical dependency and therefore the order is not arbitrary (e.g., the study design is supposed to be the starting point and reporting to be the last phase).

**Fig. 3:**
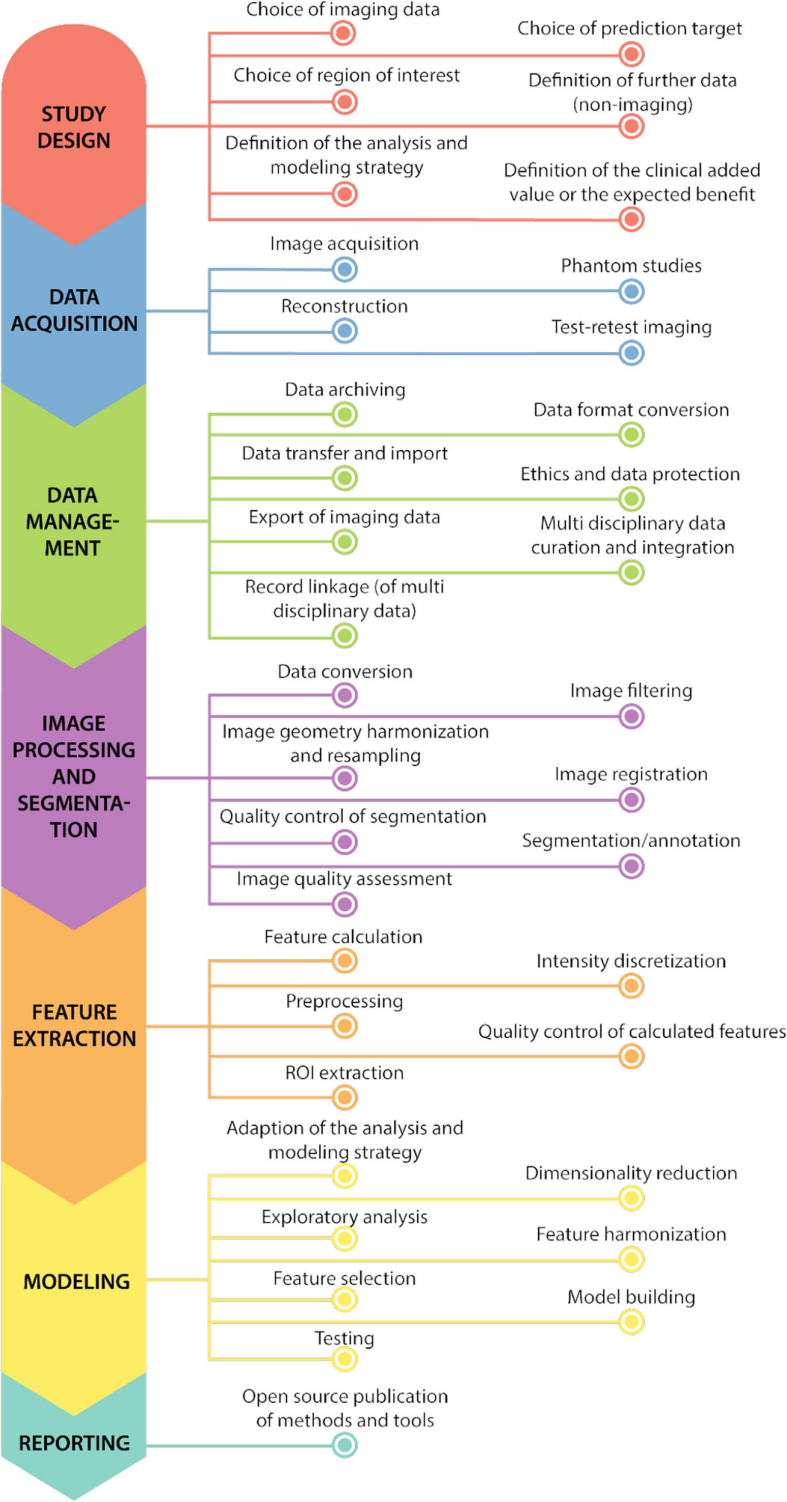
The figure shows all phases and aspects of the consensus workflow definition. The phases are shown on the left side in their logical sequence (from top to bottom). The associated aspects are shown on the right side. The aspects are sorted alphabetically and their indention is just for better readability

A phase may contain one or more aspects. Aspects are activities that take place within a phase. Aspects are often optional and have, per se, no fixed order of execution or count, as these can be highly study-specific. In the presented version of the definition, 37 aspects were defined (taken from literature or defined in the Delphi process). The phases and their aspects are depicted in Fig. 3.

Even though the overall finalization of the definition reached a high consensus (89.7% agree or strongly agree vs. 7.7% disagree or strongly disagree; 2.6% neither agree nor disagree), two aspects remain controversial. First, the question of whether the aspect “Data format conversion” should be kept separately (60%) or merged with the aspect “Data transfer and import” (33.3%) could not be answered conclusively. Second, the aspect “Image quality assessment” was discussed very controversially regarding phase association (35.9% “Image processing and segmentation” phase vs 53.9% “Data management” phase; 10.3% neither agree nor disagree) and obligation (43.6% mandatory vs 33.3% optional; 23.1% neither agree nor disagree).

The detailed version of the workflow definition (comprising names and descriptions in English and German language; compulsoriness; machine learning applicability) can be found in supplement 2 (Consensus Radiomics Workflow Definition) and a first proposal for a formal representation as an OWL ontology (W3C Web Ontology Language; https://www.w3.org/TR/2004/REC-owl-features-20040210/) will be made publicly available through https://github.com/MIC-DKFZ/radiomics-workflow-definition. In addition, we provide supplement 3, a mapping table between the consensus definition and analyzed literature terms to support the translation between terms used in different publications. We included the radiomics standardization guidelines ARISE [10], CLEAR [9], IBSI [8], and RQS [7] in this mapping and observed that only the phase “Feature extraction” is represented in all four guidelines. On average 3 of these 4 guidelines are mapping to aspects of the seven defined consensus phases, but no aspect is covered by all of the guidelines.

### Challenge characterization (stage 3)

The ten most important challenges regarding the clinical application of radiomics workflows and the perspective of the participating experts, as identified by the consensus process, are shown in Table 2. Those challenges consist of four challenges proposed by the expert panel (importance rank #2, #3, #4, and #7) and six that were derived from the screened literature (importance rank #1, #5, #6, #8, #9 and #10). From the initial seven challenge categories, five are represented in this list (A Lack of guidelines, B Lack of standardization, C Problems related to radiomics studies, D Problems related to radiomics pipelines, G Problems related to data sharing). A detailed list of all categories and challenges is provided in supplement 4 (List of challenges).

**Table 2:** Challenge importance ranking regarding clinical translation after the consensus process (displaying the 10 highest ranked challenges) (see supplement 3 - List of Challenges)

#	Challenge	Importance agreement	Category
1	Problems related to reproducibility/generalizability	51.4% (18)	Problems related to radiomics studies
2	Problem related to uncertainty/trustability of models *[expert proposal]*	40.0% (14)	Problems related to radiomics pipelines
3	Lacking workflow integration *[expert proposal]*	31.4% (11)	Problems related to radiomics pipelines
4	Lack of evidence gained by prospective evaluation *[expert proposal]*	28.6% (10)	Problems related to radiomics studies
5	Legal and privacy problems	28.6% (10)	Problems related to data sharing
6	Problems related to use of routine data	25.7% (9)	Problems related to radiomics studies
7	Lack of quality ensuring guidelines for reviewers (and editors) *[expert proposal]*	20.0% (7)	Lack of guidelines
8	Lack of standardized computation methods	17.1% (6)	Lack of standardization
9	Lack of homogeneous evaluation criteria	11.4% (4)	Lack of standardization
10	Problems related to image acquisition	11.4% (4)	Problems related to radiomics pipelines

Column “#” displays the final importance rank. Column “Importance agreement” displays the percentage of experts who picked the challenge as important, and in brackets the absolute number of selections. Challenges proposed by the expert panel in round 3 are marked by “*[expert proposal]*”

**Fig. 4:**
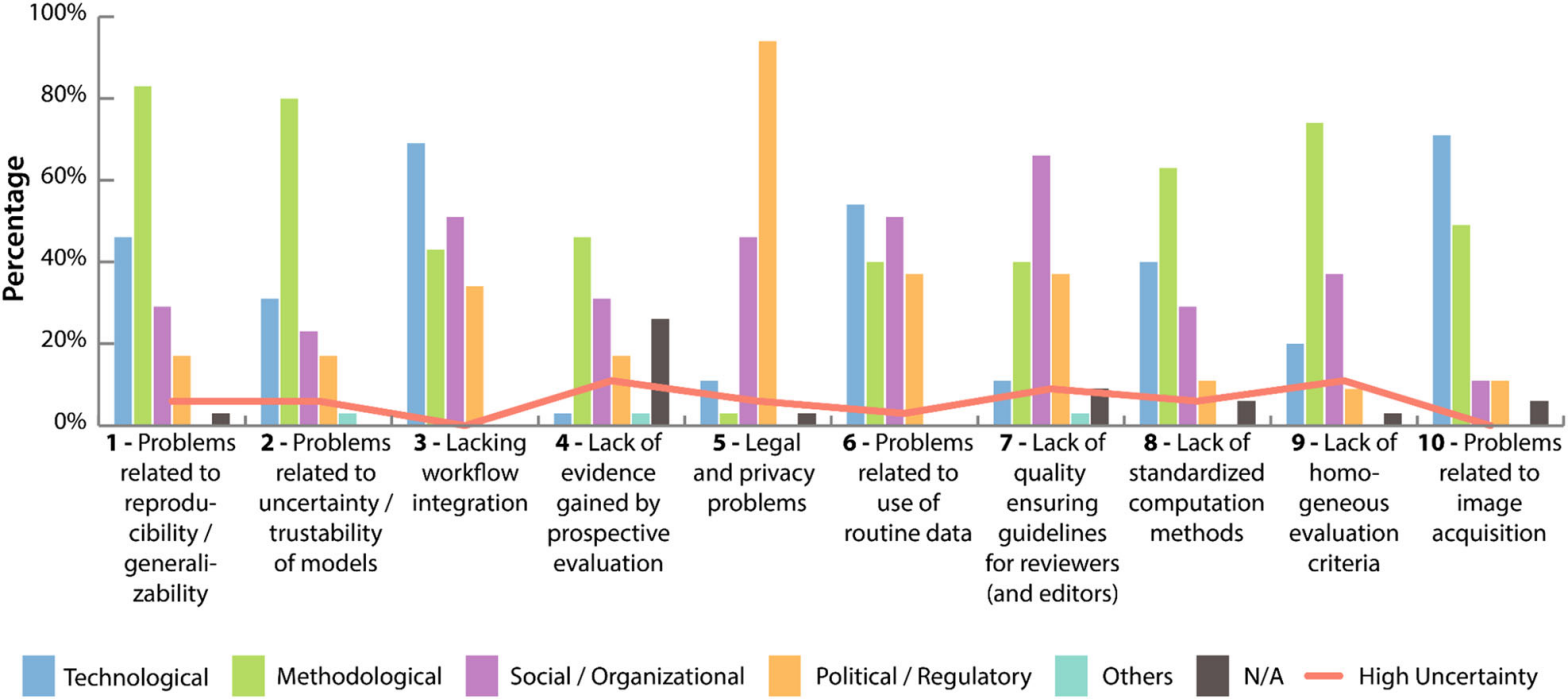
The figure shows for each challenge the percentage of experts anticipating a specific solution domain (technological, methodological, social/organizational, political/regulatory, others, and N/A) as relevant. Experts were allowed to choose multiple domains as relevant. In addition, for each challenge the percentage of experts indicating high uncertainty with regard to their selection is provided

Besides importance, the experts also rated the relevance of different solution domains to address the respective challenge. Each expert was allowed to choose multiple domains (technological; methodological; social/organizational; political/regulatory; others; N/A) and in addition could indicate high uncertainty about their response. The details of this characterization are displayed in Fig. 4. In general, most challenges were anticipated to require solutions that strongly involve multiple domains. Only three challenges (#1 and #2: methodological; #7: political/regulatory) were anticipated to have a clear domain focus (one domain > 80%).

**Fig. 5:**
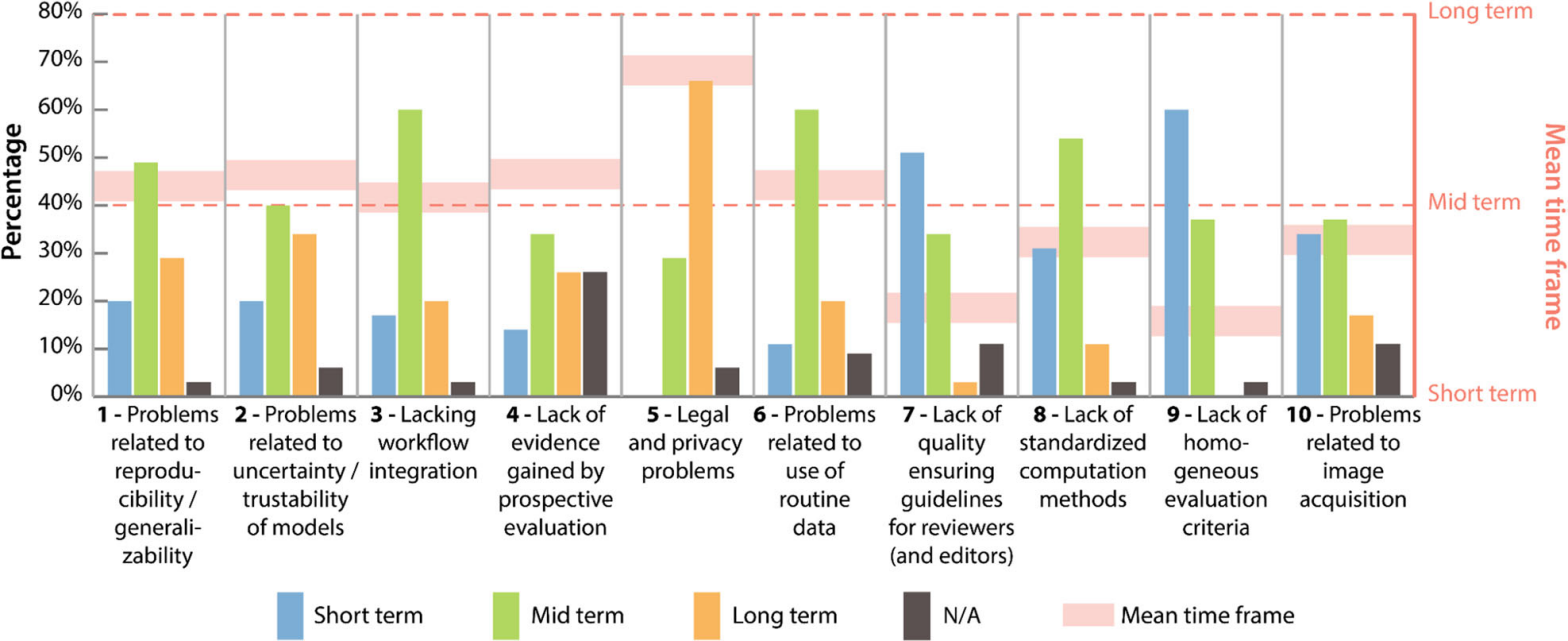
The figure shows for each challenge the anticipated time frame (short-term (≤ 2 years), medium-term (≤ 5 years), long-term (> 5 years), and N/A) to meet the respective challenge. Experts had to choose one time frame. The light red boxes show the “mean” anticipated time frame (excluding N/A selections) for each challenge

The challenges were further characterized by the anticipated time frame required to overcome them. Each expert was allowed to choose one of the following categories: short-term (≤ 2 years), medium-term (≤ 5 years), long-term (> 5 years) and N/A. Figure 5 shows the distribution of anticipated timeframe categories. Most challenges were assumed to have medium-term solution time frames. Two challenges (#7 and #9) were anticipated to be short-term and challenge #5 was anticipated to be long-term.

## Discussion

We conducted an analysis of existing radiomics workflow definitions followed by a Delphi process to achieve consensus on a common workflow definition (including an ontology) and identify the participating experts’ ten most important translation-hindering challenges. The review revealed controversial/ambiguous definitions and semantic conflicts (in total 45) in the 22 workflow definitions of the screened publications. That supports the need of a standardized workflow definition based on a broad consensus. Via the Delphi process, we achieved a radiomics workflow definition proposal with high consensus (89.7% agree or strongly agree). Further, the Delphi process allowed us to identify the challenges that were deemed most pressing by the participating experts.

Our results support the hypothesis that, while important endeavors to improve clinical translation such as IBSI, RQS, QIBA, or guideline framing are underway, there currently exists no consensus on standardized workflow definitions. Most analyzed papers include aspects of the defined consensus phases “Modelling” (96%, 23 publications) and “Feature extraction” (92%, 22 publications). Looking only at well-known radiomics standardization guidelines (ARISE [10], CLEAR [9], IBSI [8], and RQS [7]) the phase coverage overlap improves (all phases are covered to some extent by at least 3 guidelines; “Feature extraction” is covered by all). But even in this focused set of analyzed literature, the lack of definition overlap becomes evident as none of the consensus aspects is covered by all 4 guidelines. Therefore, such a standardized definition and common terminology would also support translation as it allows, i.a., a better comparability of radiomics studies. Further, such a definition would directly help to tackle two identified top challenges (#1 reproducibility/generalizability and #3 workflow integration). Some challenges have previously been addressed, e.g., IBSI addresses challenges #8 and #9. Nevertheless, the top five challenges are currently not sufficiently addressed; neither is challenge #7 (guidelines for reviewers). We would like to emphasize that our finding that IBSI addresses challenges of “lower” importance does not imply wrong targeting by IBSI. On the contrary, we see it as an indicator of the effectiveness and importance of efforts such as IBSI, as the challenges it addressed became less pressing over the last years, which resulted in lower ranks in our study.

This study has limitations. Our definition of screening is not a systematic review as it was limited by the stopping criterion employed. However, these limitations proved irrelevant for the purpose of our study. The screening served to (i) determine the presence of controversial/ambiguous definitions and (ii) provide input for the baselines of the Delphi process. Both aims were sufficiently met with the analyzed literature.

Furthermore, our team of experts was geographically limited to Germany, as they were recruited from the SPP 2177. Nevertheless, as shown in the results section, they covered a broad range of scientific fields and expertise in radiomics. Therefore, we don’t expect relevant biases in the definition consensus, but assume them more likely in the challenge prioritization. This is due to high regulatory requirements and other factors in Germany which might lead to higher prioritization of data availability and data protection challenges by our expert panel compared to experts coming from countries with less restrictive conditions. Moreover, this study represents the first consensus on workflow definition. We envision it to be a starting point for a larger community process that would address these issues. Also, for future applications, the scope of the proposed definition could be too narrow, as the consensus process began with a focus on rather classical radiomics workflows to build image feature-based prediction models. However, consensus definitions for (i) workflows that do not focus on model building but on model application (inferencing) and (ii) emerging machine learning (ML)-based workflows are missing. The former has not been addressed yet and represents a desirable goal for future iterations of the definition. The latter is covered only briefly. These ML-based approaches are only emerging and therefore, their role in a radiomics workflow is not settled yet [33, 34]. They might replace individual aspects of our consensus or, in the case of an end-to-end approach, even entire sequences of workflow phases. Therefore, we limited our scope to only indicating which phases and aspects could, given sufficient methodological progress, potentially be replaced by ML. Nevertheless, as stated above, further revisions of this consensus might address ML approaches in more depth.

Even after multiple rounds in the Delphi process, not all aspects have reached consensus yet. As the current version already offers significant value due to an overall very high consensus rate, and we envision further iterations with a larger expert panel in the future, we decided to make the remaining controversies transparent and publish the current status to initiate a broader scientific discussion.

In summary, we identified and ranked the ten most important challenges in translating radiomics into the clinic from the perspective of the participating experts. We further propose a standardized definition of terms describing phases of radiomics workflows consisting of seven major phases and 37 associated aspects that achieved high consensus among our experts. This standardized definition (supplement 2) is provided with a translation table (supplement 3) that maps the terms against the analyzed literature. As the results of this study are seen as a starting point for further developments and a broader international consensus discussion, this definition (and ontology) is publicly available online. We have prepared the resources for a future open structured definition development process (https://github.com/MIC-DKFZ/radiomics-workflow-definition) and experts from outside our network are very welcome to adapt, contribute to this, and make it their own. Standardizing the terminology in radiomics workflows can only constitute a first step towards clinical translation, with further research addressing major challenges and roadblocks urgently required. The SPP 2177 is committed to building upon the results of this study to address these challenges. By providing a common ontology for radiomics workflow definitions and identifying which challenges should be targeted with the highest priority, the presented study serves as an important foundation for future advances in the field.

### Supplementary information

Electronic Supplementary Material

## Data Availability

All data generated or analyzed during the study are included in the published paper (see supplements) or in the repository: https://github.com/MIC-DKFZ/radiomics-workflow-definition.

## Abbreviations

ARISE: Assessment of radiomics research
CLEAR: Checklist for evaluation of radiomics research
DFG: Deutsch Forschungsgemeinschaft; German Research Foundation
FAIR: FAIR principles (www.go-fair.org); Findable accessible interoperable reusable
IBSI: Image biomarker standardization initiative
ML: Machine learning
QIBA: Quantitative imaging biomarkers alliance
RQS: Radiomics quality score
SPP: Schwerpunktprogramm; Priority program
