# Supplementary material for: Radiomics workflow definition & challenges - German priority program 2177 consensus statement on clinically applied radiomics
Source: Insights Imaging. 2024 Jun 3;15:124. doi: 10.1186/s13244-024-01704-w (PMC11144687; doi:10.1186/s13244-024-01704-w)
Supplement: Supplementary file 1 — Electronic Supplementary Material [file 13244_2024_1704_MOESM1_ESM.pdf]

Radiomics Workflow Definition & Challenges - German Priority Program 2177 Consensus Statement on Clinically Applied Radiomics

ELECTRONIC SUPPLEMENTARY MATERIAL

Insights Imaging (2024) Floca R, Bohn J, Haux C, et al.

Supplement 1

SPP Radiomics – List of extracted step terms

A SPP Radiomics Workflow Definition Supplement

Version: 2024/03/09

Scope: This is a supplement for the publication "Radiomics Workflow Definitions and Challenges of Implementation in Clinics: a Delphi-based Interdisciplinary Consensus" by the Scientific Priority Program Radiomics (DFG SPP2177) by the Germany Research Foundation. The supplement contains the list of step terms extracted from analyzed literature and used for compiling the baseline for the Delphi process described in the publication.

Table of content

Table instructions 3
Table of step terms 4
Terminology conflicts 14
Literature 16
Legend 18
Terminology 19
Remarks 19
Workflow Definition 20

Table instructions

Purpose of the provided table is to illustrate the terms, found in the analyzed literature, and their relation. Further this table documents which term was used to represent a certain definition for the Delphi process and the consensus term that was finally chosen for a definition.

How to read the table

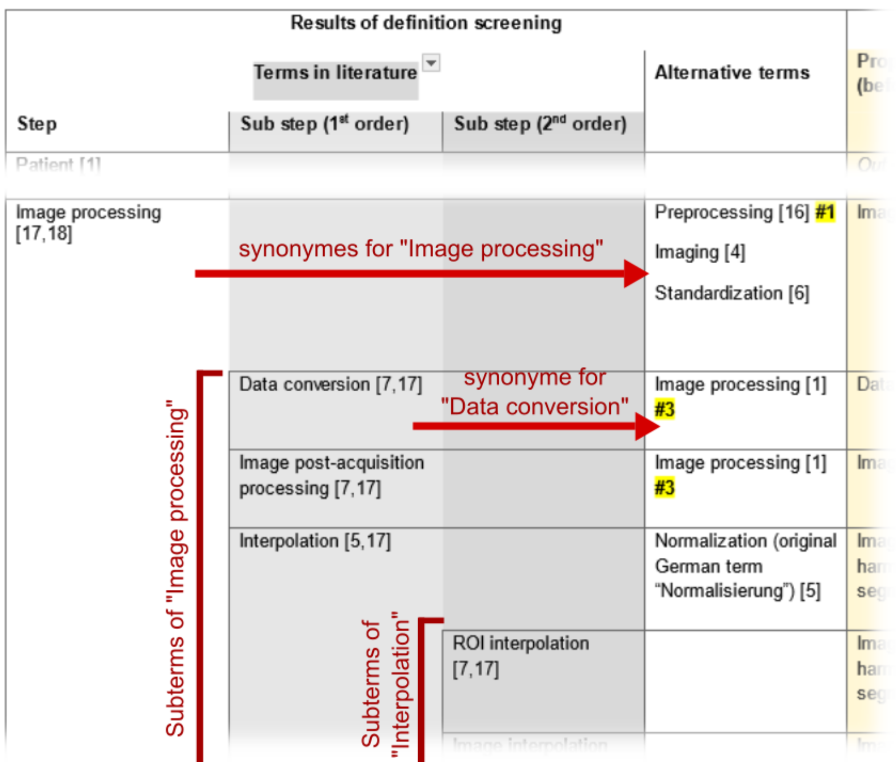

The first four columns (heading "Results of definition screening") are containing terms extracted from the analyzed literature. The first three columns (heading "Terms in literature") are needed to depict the identified hierarchies between the terms. A cell of a higher order column can span over multiple rows of the lower order column (e.g. column 1 over column 2). This indicates that all rows of the lower order column contain sub terms. E.g. the "Image processing" cell spans over multiple rows of the 2nd column which indicates that e.g. "Data conversion" and "Interpolation" are sub terms of "Image processing" (see figure on the left). In the same way it is indicated that "ROI interpolation" is a sub term of "Interpolation" (whose cell spans over the "ROI interpolation" row). The fourth column (heading "Alternative terms") contains all found synonyms for the term on the left side of the same row (e.g. "Preprocessing" is one found synonym for "Image processing"; see figure on the left).

The last two columns (heading "Delphi process") indicate which terms were used in the therapy process after the analyzes. The fifth column (heading "Proposal (before Delphi)") shows the terms that are used at the beginning of the process. The sixth column (heading "Workflow consensus (after Delphi)") shows the respective consensus terms after the Delphi process.

Remark 1: The rows are ordered according to the analysis process that constructed the tree of step terms. Therefore, the order of the last column ("Workflow consensus (after Delphi)") does not represent the structure/organization (phases and their aspects) of the consensus definition.

Remark 2: There is no dedicated meaning for the selection of which terms should be in the first three columns and which terms are declared their respective synonyms, as all synonymous terms are equivalent.

Table of step terms

Results of definition screening				Delphi process	
Terms in literature			Alternative terms	Proposal (before Delphi)	Workflow Consensus (after Delphi)
**Step**	**Sub step (1st order)**	**Sub step (2nd order)**			
Patient [1]				*Out of scope*	*Out of scope*
Data selection [2]			Creation of a dataset with appropriate clinical and radiological data [3] **#3**	Data selection	Study design<br>*(Merged into)*
	Choice of imaging protocol [2] **#3**			Choice of imaging protocol	Choice of imaging data
	Choice of prediction target [2]			Choice of prediction target	Choice of prediction target
	Choice of volume of interest [2]		ROI definition [4] #1	Choice of volume of interest	Choice of region of interest

Data acquisition (original German term “Datenakquisition”) [5]			Creation of a dataset with appropriate clinical and radiological data [3] **#3**<br><br>Medical imaging [2]<br><br>MR Image acquisition with a standardization [6]<br><br>Medical imaging acquisition [7]<br><br>Image acquisition [3,7–12]	Data acquisition	Data acquisition
	Standardization [4,6]			Choice of imaging protocol	Choice of imaging data
	Imaging at multiple time points [2]		Test-retest repeatability [1]	Test-retest imaging	Test-retest imaging
	Image acquisition [1,2,13–15]			Image acquisition	Image acquisition
	Reconstruction [1,13,15]			Reconstruction	Reconstruction
	Phantom studies [2]			Phantom studies	Phantom studies

Data management (original German term "Datenmanagmement") [5]				Data management	Data management
	Export of DICOM images from PACS to the computer software that will be used to perform the radiomics analysis [3] **#3**			PACS export	Export of Imaging Data
Image processing [17,18]			Preprocessing [16] **#1**<br><br>Imaging [4]<br><br>Standardization [6]<br><br>Computation of radiomics features [7] #3<br><br>Data preprocessing (original German term "Datenvorverarbeitung") [5]	Image processing	Image processing and segmentation<br><br>*(Merged into)*
	Data conversion [7,17]		Image processing [1] **#3**	Data conversion	Data conversion
	Image post-acquisition processing [7,17]		Image processing [1] **#3**	Image filtering	Image filtering

	Interpolation [5,17]		Normalization (original German term “Normalisierung”) [5]	Image geometry harmonization (pre segmentation)	Image geometry harmonization and resampling<br>*(Merged into / unified as)*
		ROI interpolation [7,17]		Image geometry harmonization (post segmentation)	Image geometry harmonization and resampling<br>*(Merged into / unified as)*
		Image interpolation [7,17]		Image geometry harmonization (post segmentation)	Image geometry harmonization and resampling<br>*(Merged into / unified as)*
	Analysis of outliers (original German term “Analyse von Ausreißern”) [5]			Outlier analysis	Image quality assessment

Segmentation [1,2,4,8,9,11–13,17]			Image segmentation [3,18–20]<br><br>Nodule volume segmentation [10]<br><br>Segmentation (original German term “Segmentierung”) [5]<br><br>ROI Extraction (original German term “Extraktion der ROI”) [5] **#1**<br><br>Region of interest segmentation [15,16]<br><br>Tumor labeling [6]<br><br>Specification of ROIs [14]<br><br>ROI segmentation [7]<br><br>ROI definition [6] **#1**<br><br>Computation of radiomics features [7] #3	Segmentation	Segmentation/ annotation<br><br>*(Merged into Image processing and segmentation as)*
	Re-segmentation [17]		ROI resegmentation [7]	Re-segmentation	Quality control of segmentation

Feature extraction [2,3,6,9–14,16,18,20]			Feature extraction and quantification [15]<br><br>Feature selection and extraction [8]<br><br>Radiomics feature extraction [19]<br><br>Calculation of the radiomics characteristics (original German term “Berrechnung der Radiomics-Merkmale”) [5]<br><br>Computation of image biomarker [1]<br><br>Generation of radiomics features [4]<br><br>Computation of radiomics features [7] #3	Feature extraction	Feature extraction
	Feature calculation [17]				Feature calculation
	ROI extraction [7,17] **#1**			ROI extraction	ROI extraction
	Intensity discretization [17]		Discretisation [7]	Intensity discretization	Intensity discretization

	Preprocessing [12,13] **#1**			Preprocessing	Preprocessing
Development of database [4]			Data handling [16]	Multimodal data integration	Multi disciplinary data curation and integration<br>*(+ moved to Data management)*
Modeling [1,2,21]			Statistical analysis and machine learning [7]<br>Analysis [10,11,13,16]<br>Model building [9] **#2**<br>Building predictive and prognostic models [15]<br>Statistical analysis [6,16]<br>Machine learning for building radiomics classifiers [9]<br>Analysis of database [4]	Modeling	Modeling
	Feature harmonization [22]			Feature harmonization	Feature harmonization

	Feature selection [2,3,9,11,12,14,15,18, 22]		Feature selection and extraction [8]<br><br>Radiomic feature selection [19]<br><br>Selection of radiomics characteristics (original German term “Auswahl von Radiomics-Merkmalen”) [5]<br><br>Radiomics signature modeling [20]	Feature selection	Feature selection
		Dimensionality reduction [22]	Dimension reduction [18]	Dimensionality Reduction	Dimensionality Reduction
		Exploratory analysis [2]		Exploratory analysis	Exploratory analysis
	Choice of modeling methodology [2]			Choice of modeling methodology	*Splitted into*<br><br>Definition of the analysis and modeling strategy<br><br>*and*<br><br>Adaption of the analysis and modeling strategy

	Model building [12,14,19] **#2**		Creation and application of the radiomics model (original German term “Erstellung und Anwendung des Radiomics-Modells”) [5]<br><br>Model development (original German term “Modellentwicklung”) [5]<br><br>Model construction [22]<br><br>Model training [18]<br><br>Radiomics signature modeling [20]<br><br>Multivariate Analysis and Model Building [6]	Model building	Model building
		Classification [15,22]		Classification	*Merged into* Modeling *as it is just one of several specific task types (e.g. also detection)*
	Validation [2,3,9,12,16,22]		Model analysis [20]<br><br>Performance evaluation [12]	Validation	Testing<br><br>*(renamed as validation in ML normally means the optimization of the architectural model/hyperparameter and*

					Testing is semantically covering what the publication wanted to express)
		MVI Prediction Model Evaluation [19]		Validation	Testing
		Nomogram Construction and Evaluation [19]		Validation	Testing
Reporting open-access scientific data [2]				Reporting	Reporting
Clinical application of radiomics [7]			Deployment (original German term "Bereitstellung") [5]	*Out of scope*	*Out of scope*
	Prospective evaluation of model [7]			*Out of scope*	*Out of scope*
	Personalized treatment [7]			*Out of scope*	*Out of scope*
Radiomics signature [4]				*Out of scope*	*Out of scope*

Legend:

(x): Reference to the publication using that term

#1: Term involved in a conflict of type "Homonym"

#2: Term involved in a conflict of type "Hierarchy conflict"

#3: Term involved in a conflict of type "Semantic ambiguity"

Insights Imaging (2024) Floca R, Bohn J, Haux C, et al.

Terminology conflicts

Explanation of all conflicts found while screening the workflow definitions in literature.

Synonyms: This type of conflict is indicated if a cell in column “Alternative names in literature” is not empty; each alternative term, not indicated as another type of conflict, is a synonym. The cell then contains all found synonyms for the respective term in the same row (e.g. term “Choice of volume of interest” has the synonymous term “ROI definition”). Remark: Synonyms are regarded as conflicts in this context, as it makes the “interoperability” between different publications harder as the reader has to translate between different terms.

Occurrences: 55 (including 9 synonymous usages that are also involved in other conflicts)

Homonyms: Homonyms were found when identically named steps were defined differently. This type of conflict is indicated by a term being present in multiple rows of the first 4 columns (“Result definition screening” columns).

Occurrences:

1. ROI Extraction: Used as synonym for “Segmentation” [5] and as its own term [1].
2. Preprocessing: Used as synonym for “Image processing” [16] and as its own term [12, 13] as a sub step of “Feature extraction”.
3. ROI Definition: Used as synonym for “Choice of volume of interest” [4] and as synonym for “Segmentation” [6].

Hierarchy conflicts: This type of conflict is a subclass of Homonyms. They occurred when a step was mentioned as a step in one publication, while it was a sub step in another publication. It is indicated by the same term occurring on multiple levels (nth order sub steps) of the same step term.

Occurrences:

1. Model building: Used as synonym for “Modelling” [9] and as its sub step [12,14,19].

Semantic ambiguity: Semantic ambiguities occurred where definitions of a publication could not be clearly assigned to one step, but to multiple main steps.

Occurrences:

1. Choice of imaging protocol [2]: Could be partly a sub step of “Data selection” (planning part) and partly of “Data acquisition” (execution).
2. Image processing [1]: Could be partly sub step “Data conversion” [17] or sub step “Image post-acquisition processing” [17].
3. Export of DICOM Images from PACS [3]: Could be partly a sub step of “Development of database” [4] or sub step of “Data management” [5].
4. Creation of a dataset with appropriate clinical and radiological data [3]: Could partly be “Data selection” [2] or partly “Data acquisition” [5].
5. Computation of radiomics features [7]: Could partly be “Image Processing” [17], partly “Segmentation” [1] and partly “Feature Extraction” [2].

Literature

List of literature that was analyzed in the definition screening.

1. Zwanenburg A. Radiomics in nuclear medicine: Robustness, reproducibility, standardization, and how to avoid data analysis traps and replication crisis. Eur J Nucl Med Mol Imaging. 2019;46(13):2638. doi: 10.1007/s00259-019-04391-8

2. Lambin P, Leijenaar RTH, Deist TM, et al. Radiomics: The bridge between medical imaging and personalized medicine. Nat Rev Clin Oncol. 2017;14(12):749. doi: 10.1038/nrclinonc.2017.141

3. Horvat N, Bates DDB, Petkovska I. Novel imaging techniques of rectal cancer: What do radiomics and radiogenomics have to offer? A literature review. Abdom Radiol (NY). 2019;44(11):3764. doi: 10.1007/s00261-019-02042-y

4. Scheckenbach K. Radiomics: Big data instead of biopsies in the future Laryngorhinootologie. 2018;97(S 01):S114. doi: 10.1055/s-0043-121964

5. Murray JM, Kaissis G, Braren R, Kleesiek J. Wie funktioniert radiomics. Radiologe. 2020;60(1):32. doi: 10.1007/s00117-019-00617-w

6. Chaddad A, Kucharczyk MJ, Daniel P, et al. Radiomics in glioblastoma: Current status and challenges facing clinical implementation. Front Oncol. 2019;9:374. doi: 10.3389/fonc.2019.00374

7. Vallières M, Zwanenburg A, Badic B, Cheze Le Rest C, Visvikis D, Hatt M. Responsible radiomics research for faster clinical translation. J Nucl Med. 2018;59(2):189. doi: 10.2967/jnumed.117.200501

8. Hassani C, Varghese BA, Nieva J, Duddalwar V. Radiomics in pulmonary lesion imaging. AJR Am J Roentgenol. 2019;212(3):497. doi: 10.2214/AJR.18.20623

9. Avanzo M, Stancanello J, El Naqa I. Beyond imaging: The promise of radiomics. Phys Med. 2017;38:122. doi: 10.1016/j.ejmp.2017.05.071

10. Wilson R, Devaraj A. Radiomics of pulmonary nodules and lung cancer. Transl Lung Cancer Res. 2017;6(1):86. doi: 10.21037/tlcr.2017.01.04

11. Lambin P, Rios-Velazquez E, Leijenaar R, et al. Radiomics: Extracting more information from medical images using advanced feature analysis. Eur J Cancer. 2012;48(4):441. doi: 10.1016/j.ejca.2011.11.036

12. Ibrahim A, Vallières M, Woodruff H, et al. Radiomics analysis for clinical decision support in nuclear medicine. Semin Nucl Med. 2019;49(5):438. doi: 10.1053/j.semnuclmed.2019.06.005

13. Fornacon-Wood I, Faivre-Finn C, O'Connor JPB, Price GJ. Radiomics as a personalized medicine tool in lung cancer: Separating the hope from the hype. Lung Cancer. 2020;146:197. doi: 10.1016/j.lungcan.2020.05.028

14. Lee S-H, Park H, Ko ES. Radiomics in breast imaging from techniques to clinical applications: A review. Korean J Radiol. 2020;21(7):779. doi: 10.3348/kjr.2019.0855

15. Thawani R, McLane M, Beig N, et al. Radiomics and radiogenomics in lung cancer: A review for the clinician Lung Cancer. 2018;115:34. doi: 10.1016/j.lungcan.2017.10.015

16. Machicado JD, Koay EJ, Krishna SG. Radiomics for the diagnosis and differentiation of pancreatic cystic lesions. Diagnostics (Basel). 2020;10(7):505. doi: 10.3390/diagnostics10070505

17. Zwanenburg A, Vallières M, Abdalah M, et al. The image biomarker standardization initiative: Standardized quantitative radiomics for high-throughput image-based phenotyping. Radiology. 2020;295(2):328. doi: 10.1148/radiol.2020191145

18. van Timmeren JEs, Cester D, Tanadini-Lang S, Alkadhi H, Baessler B. Radiomics in medical imaging—“how-to” guide and critical reflection. Insights Imaging. 2020;11(1):91. doi: 10.1186/s13244-020-00887-2

19. Yang L, Gu D, Wei J, et al. A radiomics nomogram for preoperative prediction of microvascular invasion in hepatocellular carcinoma. Liver Cancer. 2019;8(5):373. doi: 10.1159/000494099

20. Gu D, Hu Y, Ding H, et al. CT radiomics may predict the grade of pancreatic neuroendocrine tumors: A multicenter study. Eur Radiol. 2019;29(12):6880. doi: 10.1007/s00330-019-06176-x

21. Moons KGM, Altman DG, Reitsma JB, et al. Transparent reporting of a multivariable prediction model for individual prognosis or diagnosis (TRIPOD): Explanation and elaboration. Ann Intern Med. 2015;162(1):W1. doi: 10.7326/M14-0698

22. Mayerhoefer ME, Materka A, Langs G, et al. Introduction to radiomics. J Nucl Med. 2020;61(4):488. doi: 10.2967/jnumed.118.222893

Supplement 2

SPP Radiomics – Consensus Radiomics Workflow Definition

A SPP Radiomics Workflow Definition Supplement

Version: 1.0 – 2024/03/15

Scope: This is a supplement for the publication “Radiomics Workflow Definitions and Challenges of Implementation in Clinics: a Delphi-based Interdisciplinary Consensus” by the Scientific Priority Program Radiomics (DFG SPP2177) by the Germany Research Foundation. The supplement contains the consensus definition after the delphi process described in the publication. It also reflects all accepted additions and refinements proposed by the expert panel as well as the aspects that remain controversial. In addition it provides a mapping table between the consensus definition and analyzed literature.

Legend

Consensus color encoding:

Consensus:<br>>75% agreement (Agree or Strongly Agree)	
Minor controversy<br>>55% / <75% agreement (Agree or Strongly Agree)	(yellow)
Major controversy<br><55% agreement (Agree or Strongly Agree)	(red)

Phases: ***Are printed italic and bold.***

Aspects: Are printed in normal text.

Terminology

- Workflow: A workflow is structured in phases and their aspects and comprises any activity/step, to plan, to conduct and to report the building of an image feature-based prediction model.
- Phase: Phases represent different fundamental workflow steps and therefore can be found to a certain extent in every Radiomics workflow. A phase may contain one or more aspects. Between phases there is a logical dependency and therefore the order is not arbitrary.
- Aspects: Aspects are activities that take place within a phase. Aspects are often optional and they have per se no fixed order (sorting in this document is alphabetically by the English name of the aspect). As an effect of this ambiguity, the literature partially strongly differs on the aspects and their sequence. In some cases aspects are arranged in hierarchies (e.g. because we found sub aspects in the literature).

Remarks

- Mandatory/optional workflow elements: As a default, aspects of a workflow can be regarded as optional (in occurrence, order or number). The list below compiles aspects that are documented in literature and are commonly found in Radiomics workflows. Nevertheless, we think that some aspects are crucial in the Radiomics workflow to ensure the validity and reliability of its results. Those “mandatory” aspects are indicated in the column “Mandatory”. Workflow phases that have at least one mandatory aspect are also mandatory.
- Machine learning / Deep learning: Deep learning techniques are increasingly applied also in the context of Radiomics workflows. They are (potentially) applicable at many aspects of the workflow; from simply replacing single aspects (like doing the annotation) up to replacing large parts of the workflow (e.g. end-to-end approaches). Therefore, the usage of deep learning techniques in the context of the workflow definition is not represented by additional optional aspects (which would be highly redundant) but by indicating which aspects can be replaced/covered by a deep learning technique (indicated by the column “ML”).

Workflow Definition

English Name	German Name	Definition (English)	Mandatory	ML
Study design	***Studiendesign***	***Definition of the (clinical) research question, the imaging data and other data required as well as the analysis strategy. The definition should precede the analysis and always serves to ensure a reproducible and representative analysis.***	X	
Choice of imaging data	Definition der Bildgebungsdaten	Definition of image data / standardization of the imaging protocol to ensure the feasibility and reproducibility of the analysis.	X	
Choice of prediction target	Definition des Prädiktionsziels	Definition of the prediction goal of the model. (e.g. stratification with respect to progression-free survival).	X	
Choice of region of interest	Definition der Zielstruktur	Definition of the structures (ROI) to be analyzed incl. the segmentation protocol.	X	
Definition of further data (non-imaging)	Definition weiterer Daten (keine Bildgebungsdaten)	Specification of the non-imaging data used for modeling. The data can be both modeling features and data on relevant endpoints. Due to the often low standardization of many non-imaging data, the formats and terminologies used should also be defined in advance and the widest possible/established options for coding should be used.		
Definition of the analysis and modeling strategy	Definition der Analysestrategie	Definition of the analysis and modeling strategy to answer the defined research question with the selected data.		
Definition of the clinical added value or the expected benefit	Definitionen des klinischen Mehrwertes (Motivation) und des erwarteten Nutzens	Definition of the added clinical value or the expected benefit, which should be achieved by the created model.		

Data acquisition	***Datenakquisition***	***Summary of all activities necessary for prospective image data collection/standardization including associated metadata.***		
Image acquisition	Bildaufnahme	This aspect refers solely to the image acquisition and the associated acquisition parameters.		
Phantom studies	Phantomstudien	Use of phantom studies to calibrate imaging systems for a prospective study; especially for multi-center studies. Furthermore, phantom studies can be used to investigate differences between scanners and segmentation methods (inter-observer variability).		
Reconstruction	Rekonstruktion	Use of a reconstruction algorithm to reconstruct the image volume from the raw data.		**X**
Test-retest imaging	Test-Retest-Bildgebung	Experimental reproducibility assessment by repeating recordings with temporal delay to detect normal variations in the image signal (test retest).		
Data management	***Datenmanagement***	***This phase contains all the actions necessary to compile the study data for the analysis and make it available for processing in the radiomics pipeline.***	**X**	
Data archiving	Datenarchivierung	Archiving / Storage of data for potential re-analysis, subsequent validation or further research.<br><br>FAIR principles should be regarded/supported by the chosen archiving strategy.		
Data format conversion	Datenformat-Konvertierung	Conversion of the data into other data formats (e.g. from DICOM to NIFTI). This is solely the transformation of the format (in the case of a lossless conversion). The conversion of the actual data takes place in the "Data conversion" aspect in the "Image processing and segmentation" phase.		

Data transfer and import	Datentransfer und -import	Transfer and import of the data into a target system which is required for the execution of the workflow (e.g. the evaluation is not conducted in the same facility or is conducted in a non-integrated system).		
Ethics and data protection	Ethik und Datenschutz	Display of ethical vote, detailing on data protection means (anonymisation/ pseudonymisation) also for secondary use of the employed data.	X	
Export of Imaging Data	Export der Bilddaten	Export of Imaging Data (e.g. DICOM images) from the data archive (e.g. PACS) to be able to use them in the Radiomics pipeline.	X	
Multi disciplinary data curation and integration	Multidisziplinäre Datenkuratierung und -Integration	Optional inclusion of non image data (e.g. clinical data, genetic data) that should be used for the modeling.		
Record linkage (of multi disciplinary data)	Verknüpfung der Datensätze (multidisziplinär)	Linking/merging of data (from different primary sources; e.g. multidisciplinary data with different IDs) of a subject.		
Image processing and segmentation	***Bildverarbeitung und Segmentierung***	***This phase contains all actions necessary to create the segmentations and prepare images as well as segmentations for feature calculation.***		X
Data conversion	Datenkonvertierung	Conversion of the image signal (image data) into another representation (e.g. conversion of PET signal image into Standardized Uptake Values (SUV)).		X
Image filtering	Bildfilterung	Processing of the image signal with filters (e.g. noise reduction, gray value normalization,...).		X

Image geometry harmonization and resampling	Harmonisierung der Bildgeometrie und Resampling	Step to convert all images in the evaluation into an identical image geometry, in order to make voxel size-dependent features comparable. The harmonization can be done before the segmentation (the segmentation is thus performed on the harmonized geometries) or after the segmentation (The segmentation must therefore also be harmonized and resegmentation may be necessary).		X
Image registration	Bildregistrierung	Transfer of images to target geometries with a given mapping rule (e.g. to compensate for motion artifacts, to spatially align multimodal images or to normalize to a reference anatomy).		X
Quality control of segmentation	Qualitätskontrolle der Segmentierung	Checking and correction of segmentation (especially at its edges) to correct errors that were e.g. introduced by the segmentation or by "harmonization of image geometry" (post segmentation).		X
Segmentation/annotation	Segmentierung/Annotation	Segmentation/annotation of the defined region of interest based on the defined protocol.		X
Image quality assessment	Qualitätsbewertung der Bilddaten	The analysis of outliers is used to evaluate the quality of the used image material. For the assessment general criteria (e.g. correct modality or right body part) as well as study specific criteria (e.g. need minimal resolution, absence of artifacts) can be used. The assessment can be implemented by expert decision as well as automated quality control (to support scalability and reproducibility, it is adviseable to implement a process that is as quantitative and automated as possible.	X	X
Feature extraction	***Merkmalsextraktion***	***In this phase, all aspects are summarized that are necessary/relevant for feature extraction, i.e. the derivation of quantitative information from the segmented images using mathematical formulas.***	X	X

Feature calculation	Merkmalsberechnung	The actual process of calculating individual features based on the input data, the formula/algorithm and their parameterization.	X	X
Intensity discretization	Intensitätsdiskretisierung	A discretization/binning of intensities within the ROI is performed to make the calculation of texture features comprehensible and to suppress noise. Binning can be performed for all features or adapted for specific features.		X
Preprocessing	Vorverarbeitung	The preprocessing steps in this phase are used to prepare the images before feature extraction. In contrast to the aspect "Image Processing" (Phase Image Processing & Segmentation), "Preprocessing" only includes preprocessing steps that are needed for specific features (e.g. a Fourier transformation) but have no general relevance or validity. In the works studied, the preprocessing steps named were i.a. filtering in general, edge reduction or smoothing.		X
Quality control of calculated features	Qualitätskontrolle der berechneten Merkmale	Quality control (e.g. through automatic plausibility check or random checks) of the calculated features.		X
ROI extraction	ROI-Extraktion	Isolation of one or more of the ROIs from the rest of the image (e.g. by replacing excluded pixels/voxels with NaN). This step depends on the feature and the implementation of the extraction method.		X
Modeling	***Modellierung***	***This phase contains all aspects that are necessary to establish a model that, based on given input data (radiomic features, clinical features, etc.), allows prediction in terms of the defined prediction goal.***	x	

Adaption of the analysis and modeling strategy	Anpassung der Analyse- und Modellierungsstrategie	Sometimes it can be necessary to adapt the analysis strategy of the study in order to achieve the research goal with the given study data. This aspect covers this necessity, but should be avoided if possible. If one has to adapt the strategy and diverge from the original study design, it should be handled very carefully and statistically double-checked to ensure the validity and integrity of the results. As later changes can introduce bias, over fitting or statistical errors and alike, the results could otherwise be compromised.	**X**	
Dimensionality Reduction	Dimensionsreduktion	Combination of several features into a new feature (e.g. by Principal Component Analysis).		
Exploratory analysis	Explorative Analyse	Interactive analysis on the predictive power of different combinations of radiomic features and non-radiomic features. This can be used to perform more targeted feature selection and reduction. It is important that this aspect is not done with the data for testing.		
Feature harmonization	Merkmalsharmonisierung	Mathematical method to correct batch effects (e.g. location-dependent variations during the image acquisition). In contrast to a harmonization of the gray values (e.g. also by phantom studies, see above), here the harmonization takes place only after the extraction of the features.		
Feature selection	Auswahl von radiomischen Merkmalen	Selection of radiomic features that are relevant and informative for the planned task, from the extracted radiomic features (e.g. mRMR). Another criterion is the exclusion of non-reproducible radiomic features.		

Model building	Modellentwicklung	The optimization of a model, to ensure a best possible prediction of the prediction targets based on the selected features. This includes i.a. the parameter optimization of parameterized models (Training) or architectural model/ hyperparameter optimization (Validation).	X	
Testing	Testen	Testing using dedicated data, which is not used for model training, serves the final evaluation of the suitability of the radiomics model (e.g. with regard to robustness/generalizability, predictive quality/accuracy...). Ideally, this is done by means of an independent test set. The testing can also be done by means of cross-validation (whereby it must then be ensured that all aspects mentioned above must be cross-validated, including e.g. nested cross-validation for hyperparameter optimization).<br>Remark: The term "Testing" was preferred over often found "Validation" because validation in ML normally means the optimization of the architectural model/hyperparameter and "Testing" is semantically fitting term in ML.	X	
Reporting	***Report***	***Report of the results including all necessary metadata (data provenance, data source, processing steps, data quality) as part of publications or the enablement of subsequent usage . The FAIR principles should be regarded/implemented in the report.***	X	
Open source publication of methods and tools	Open source Publikation der Methoden und Werkzeuge	Publication (e.g. via public code repositories) of the used program source codes and analysis scripts to support reproducibility of the study setup and reuse of the published methodology. ***The FAIR principles should be regarded/implemented in the code publication.***		

Supplement 3

Consensus definition	Zwanenburg 2019 [1]	RQS Lambin 2017 [2]	Horvat 2019 [3]	Scheckenbach 2018 [4]	Murray 2020 [5]	Chaddad 2019 [6]
Study design			***Creation of a dataset with appropriate clinical and radiological data #1***			
Choice of imaging data		Choice of imaging data #2		Standardization		Standardization
Choice of prediction target		Choice of prediction target				
Choice of region of interest		Choice of region of interest		ROI definition		
Definition of further data (non-imaging)						
Definition of the analysis and modeling strategy		Choice of modeling methodolog				
Definition of the clinical added value or the expected benefit						
Data acquisition		***Medical Imaging***	***Creation of a dataset with appropriate clinical and radiological data #1; Image acquisition***		***Datenakquisition***	***MR Image acquisition with a standardization***

Image acquisition	Image acquisition	Image acquisition				
Phantom studies		Phantom studies				
Reconstruction	Reconstruction					
Test-retest imaging	Standardization	Imaging at multiple time points				
Data management		***Creation of databases; Data management***			***Datenmanagement***	
Data archiving						
Data format conversion						
Data transfer and import						
Ethics and data protection						
Export of Imaging Data			Export of DICOM images from PACS to the computer software that will be used to perform the radiomics analysis #3			

Multi disciplinary data curation and integration		Radiogenomics	Export of DICOM images from PACS to the computer software that will be used to perform the radiomics analysis #3	Development of database		
Record linkage (of multi disciplinary data)						
Image processing and segmentation		***Data selection***		***Imaging***	***Datenverarbeitung***	***Standardization***
Data conversion	Image processing #5					
Image filtering	Image processing #5					
Image geometry harmonization and resampling					Interpolation; Normalisierung	
Image registration						
Quality control of segmentation						
Segmentation/annotation	Segmentation	Segmentation	Image segmentation	Segmentation	Segmentierung Extraktion der ROI	Tumor labeling ROI definition
Image quality assessment					Analyse von Ausreißern	
Feature extraction	***Computation of image biomarker***	***Feature extraction***	***Feature extraction***	***Generation of radiomics features***	***Berrechnung der Radiomics-Merkmale***	***Feature extraction***

Insights Imaging (2024) Floca R, Bohn J, Haux C, et al.

Feature calculation						
Intensity discretization						
Preprocessing						
Quality control of calculated features						
ROI extraction						
Modeling	***Modeling***	***Modeling; Radiomics Modeling***		***Analysis of database***		***Statistical analysis***
Adaption of the analysis and modeling strategy		Choice of modeling methodolog				
Dimensionality Reduction		Dimensionalty Reduction				
Exploratory analysis		Exploratory analysis				
Feature harmonization						
Feature selection		Feature selection	Feature selection		Auswahl von Radiomics-Merkmalen	
Model building					Erstellung und Anwendung des Radiomics-Modells; Modellentwicklung	Multivariate Analysis and Model Building

Testing		Validation	Validation			
Reporting		***Reporting open-access scientific data***				
Open source publication of methods and tools						

**Consensus definition**	**Vallières 2018 [7]**	**Hassani 2019 [8]**	**Avanzo 2017 [9]**	**Wilson 2017 [10]**	**Lambin 2012 [11]**	**Ibrahim 2019 [12]**
Study design						
Choice of imaging data						
Choice of prediction target						
Choice of region of interest						
Definition of further data (non-imaging)						
Definition of the analysis and modeling strategy						
Definition of the clinical added value or the expected benefit						

Data acquisition	***Medical imaging acquisition; Image acquisition***		***Image acquisition***	***Image acquisition***	***Image acquisition***	***Image acquisition***
Image acquisition						
Phantom studies						
Reconstruction						
Test-retest imaging						
Data management						
Data archiving						
Data format conversion						
Data transfer and import						
Ethics and data protection						
Export of Imaging Data						
Multi disciplinary data curation and integration						

Record linkage (of multi disciplinary data)						
Image processing and segmentation	***Computation of radiomics features #4***					
Data conversion	Data conversion					
Image filtering	Image post-acquisition processing					
Image geometry harmonization and resampling	Image interpolation;<br>ROI Interpolation					
Image registration						
Quality control of segmentation						
Segmentation/annotation	Computation of radiomics features #4;<br>ROI segmentation	Segmentation	Segmentation	Nodule volume segmentation	Segmentation	Segmentation
Image quality assessment						
Feature extraction	***Computation of radiomics features #4***	***Feature extraction and selection***	***Feature extraction***	***Feature extraction***	***Feature extraction***	***Feature extraction***
Feature calculation						
Intensity discretization	Discretization					

Preprocessing						Preprocessing
Quality control of calculated features						
ROI extraction	ROI extraction					
Modeling	***Statistical analysis and machine learning***		***Model building; Machine learning for building radiomics classifiers***	***Analysis***	***Analysis***	
Adaption of the analysis and modeling strategy						
Dimensionality Reduction						
Exploratory analysis						
Feature harmonization						
Feature selection		Feature selection and extraction	Feature selection		Feature selection	Feature selection
Model building						Model building
Testing			Validation			Performance evaluation; Validation
Reporting						

Insights Imaging (2024) Floca R, Bohn J, Haux C, et al.

Open source publication of methods and tools						

**Consensus definition**	**Fornacon-Wood 2020 [13]**	**Lee 2019 [14]**	**Thawani 2017 [15]**	**Macchicado 2020 [16]**	**IBSI Zwanenburg 2020 [17]**	**van Timmeren 2020 [18]**
Study design						
Choice of imaging data						
Choice of prediction target						
Choice of region of interest						
Definition of further data (non-imaging)						
Definition of the analysis and modeling strategy						
Definition of the clinical added value or the expected benefit						
Data acquisition						

Image acquisition	Image acquisition	Image acquisition	Image acquisition			
Phantom studies						
Reconstruction	Reconstruction		Reconstruction			
Test-retest imaging						
Data management						
Data archiving						
Data format conversion						
Data transfer and import						
Ethics and data protection						
Export of Imaging Data						
Multi disciplinary data curation and integration				Data handling		
Record linkage (of multi disciplinary data)						

Image processing and segmentation				***Preprocessing***	***Image processing***	***Image processing***
Data conversion					Data conversion	
Image filtering					Image post-acquisition processing	
Image geometry harmonization and resampling					Interpolation; Image interpolation; ROI interpolation	
Image registration						
Quality control of segmentation					Re-segmentation	
Segmentation/annotation	Segmentation	Specification of ROIs	Region of interest segmentation		Segmentation	Image segmentation
Image quality assessment						
Feature extraction	***Feature extraction***	***Feature extraction***	***Feature extraction and quantification***	***Feature extraction***		***Feature extraction***
Feature calculation					Feature calculation	
Intensity discretization					Intensity discretization	
Preprocessing	Preprocessing					

Quality control of calculated features						
ROI extraction					ROI extraction	
Modeling	***Analysis***		***Building predictive and prognostic models; Classification***	***Analysis; Statistical analysis***		
Adaption of the analysis and modeling strategy						
Dimensionality Reduction						Dimension reduction
Exploratory analysis						
Feature harmonization						
Feature selection		Feature selection	Feature selection			Feature selection
Model building		Model building				Model training
Testing				Validation		
Reporting						
Open source publication of methods and tools						

[redacted]

**Consensus definition**	**Yang 2019 [19]**	**Gu 2019 [20]**	**TRIPOD Moons 2015 [21]**	**Mayerhoefer 2020 [22]**	**CLEAR Kocak 2023 [23]**	**ARISE Kocak 2023 [24]**
Study design					Study design	Design
Choice of imaging data					Selection of imaging modality	
Choice of prediction target						
Choice of region of interest						
Definition of further data (non-imaging)						
Definition of the analysis and modeling strategy						
Definition of the clinical added value or the expected benefit						
Data acquisition					Data acquisition	
Image acquisition					Image acquisition	
Phantom studies					Phantom studies	

Reconstruction						
Test-retest imaging						
Data management					Data preperation	
Data archiving						
Data format conversion						
Data transfer and import						
Ethics and data protection					Ethics and data protection	
Export of Imaging Data						
Multi disciplinary data curation and integration						
Record linkage (of multi disciplinary data)						
Image processing and segmentation					Segmentation strategy and Preprocessing	
Data conversion						

Image filtering					Image types	
Image geometry harmonization and resampling						
Image registration						
Quality control of segmentation						
Segmentation/annotation	Image segmentation	Image segmentation			Segmentation	
Image quality assessment						
Feature extraction	Radiomics feature extraction	Feature extraction			Feature extraction	Feature extraction/ Radiomics methodology
Feature calculation						
Intensity discretization						
Preprocessing					Preprocessing	Preprocessing
Quality control of calculated features						
ROI extraction						

Modeling			Modeling	Classification	Modeling	
Adaption of the analysis and modeling strategy						
Dimensionality Reduction				Dimensionality Reduction		
Exploratory analysis						
Feature harmonization				Feature harmonization		
Feature selection	Radiomics feature selection	Radiomics signature modeling		Feature selection		
Model building	Model building	Radiomics signature modeling		Model construction	Training and tuning	
Testing	MVI Prediction Model Evaluation; Nomogram Construction and Evaluation	Model analysis		Validation	Testing	Internal and external testing
Reporting					Reporting	Metrics and reporting
Open source publication of methods and tools					Open science	

Legend:
#1: Semantic ambiguity: Could be part of “Study design” or partly “Data acquisition”.
#2: Semantic ambiguity: Could partly be a sub step of “Study design” (planning part) and partly of “Data acquisition” (execution).
#3: Semantic ambiguity: Could partly be “Export of imaging data” and partly “Multi disciplinary data curation”.
#4: Semantic ambiguity: Could partly be “Image processing and Segmentation” and partly “Feature Extraction”.
#5: Semantic ambiguity: Could partly be “Data conversion” and partly “Image filtering”.

Supplement 4

SPP Radiomics – List of challenges

A SPP Radiomics Workflow Definition Supplement

Version: 2024/03/13

Scope: This is a supplement for the publication "Radiomics Workflow Definitions and Challenges of Implementation in Clinics: a Delphi-based Interdisciplinary Consensus" by the Scientific Priority Program Radiomics (DFG SPP2177) by the Germany Research Foundation. The supplement contains a list of all challenges regarding the translation and clinical application of radiomics extracted from literature or proposed by experts. The challenges proposed by the experts have an index marked with *.X*) and were collected in the third round of the Delphi process described in the publication.

Remark: Challenge F.1 "Lack of reproducibility of biomarkers" was merged into C.5 "Problems related to reproducibility /generalizability" for round 5 of the Delphi process as on this abstraction level F.1 can be seen as a subset of C.5.

A Lack of guidelines

This category contains challenges that originate in a lack of guidelines for important/relevant steps in the workflow.

A.1 Lack of processing guidelines

A.2 Lack of reporting guidelines

A.X1 Lack of quality ensuring guidelines for reviewers (and editors)
(e.g. stricter guidelines to ensure scientific quality and manage the increasing number of radiomics; preventing quantity rather than quality (which is pushed by "publish and perish))

B Lack of standardization

This category contains challenges that originate in a lack of standardization of aspects in the workflow .

B.1 Lack of homogenous evaluation criteria
(e.g. the way evaluations are conducted (i.a. metrics to use, baselines) is not standardized; reporting guidelines not applied)

B.2 Lack of standardized computation methods
(e.g. the feature computation is not conducted in a standardized way)

C Problems related to radiomics studies

This category contains challenges that originate in problems with the study design or the way the study is conducted.

C.1 Lack of definition of a "good" radiomics study

C.2 Problems related to use of routine data
(e.g. the routine data proofs not to be suitable to answer the research question (i.a. due to problems with quantity, quality or contained information); other problems with the data not covered elsewhere)

C.3 Problems related to study design
(e.g. Insufficient size of patient cohorts, Lack of statistical significance, Non-consistent conduct of radiomics studies, High false-positive rates, Class imbalance; Insufficient / irrelevant clinical contribution (problem addressed is not relevant in clinical routine, e.g. radiomics prediction of IDH mutation status in gliomas))

C.4 Problems related to study results
(e.g. Lack of validation of studies, Causality difficult to establish, Overly optimistic results, Problems related to reporting of studies, Insufficient reporting about patient data, Insufficient reporting about prediction models, Insufficient reporting about methodological information)

C.5 Problems related to reproducibility /generalizability
(e.g. Lack repeatability, Lack of generalizability, Lack of reproducibility of studies)

C.X1 Lack of evidence gained by prospective evaluation
(e.g. performances of retrospective results cannot be reproduced in a prospective setting)

D Problems related to radiomics pipelines

This category contains challenges that originate in problems with the way the processing is done or the used tooling.

D.1 Problems related to image acquisition
(e.g. Imaging inconsistency, Imaging at multiple time points, Use of not standardized image acquisition protocols, Differences between imaging modalities, Lack of standardization of acquisition parameters, Heterogeneity caused by variations in acquisition parameters, different sequences (MRT), Contrast agent application protocol, used reconstruction kernels)

D.2 Problems related to prediction models
(e.g. Choosing a suitable algorithm for model building, Underfitting, Overfitting, Lack of generalizability of Radiomics Models, Lack of clinical utility, Lack of reproducibility of prediction models, Lack of standardized performance evaluation)

D.3 Problems related to image processing methods
(e.g. Number of subsets, Gaussian filter width for post reconstruction smoothing, unspecified hyperparameters)

D.5 Problems related to segmentations
(e.g. Differences in segmentation methods / software, Intra-/ Interobservervariability of segmentation, Reproducibility of segmentation methods)

D.6 Inconsistent processing schemes

D.7 Inconsistent radiomic methods

D.8 Variations in mathematical definitions

D.9 Differences in radiomics toolboxes

D.10 Differences between observers

D.11 Experimental inconsistency

D.X1 Problem related to uncertainty/trustability of models
(e.g. Workflows are not use because the uncertainty of their results can not be investigated; Workflows cannot detect that input is OOD and react/escalate accordingly)

D.X2 Lacking workflow integration
(e.g. Workflows are not well integrated in current workflows; imposing extra steps or systems that hinder normal workflow; too much time consumption by needed interactions)

E Problems related to data management

This category contains challenges that originate in problems with handling the data used in the study (e.g. including additional data not to the correct samples).

E.1 Complex data management

E.2 Difficulties integrating other data sources

F Problems related to radiomics features

This category contains challenges that originate in problems with the used radiomics features (e.g. use different radiomics extraction pipelines that have different names for the same features).

F.1 Lack of reproducibility of biomarkers

F.2 Variations in feature nomenclature

F.3 Lack of standardized radiomic features

F.4 Variability in radiomic feature generation

G Problems related to data sharing

This category contains challenges that originate in problems with sharing or reusing data for a study (e.g. not sharing data of private data sources makes studies not reproducible).

G.1 Legal and privacy problems
(e.g. open questions regarding model training/sharing and GPDR; Data sharing and GPDR/(broad)consent management; implications of Medical Device Directive if research software is applied prospectively (often not bearable from research groups))

G.2 Political and academic value of data

G.3 Hazards to reputation

G.4 Data recording methods

G.5 Cultural and language difficulties

G.6 Insufficient time

G.7 Insufficient human resources
